# Integration analysis of senescence-related genes to predict prognosis and immunotherapy response in soft-tissue sarcoma: evidence based on machine learning and experiments

**DOI:** 10.3389/fphar.2023.1229233

**Published:** 2023-07-11

**Authors:** Lin Qi, Fangyue Chen, Lu Wang, Zhimin Yang, Wenchao Zhang, Zhihong Li

**Affiliations:** ^1^ Department of Orthopedics, The Second Xiangya Hospital, Central South University, Changsha, China; ^2^ Hunan Key Laboratory of Tumor Models and Individualized Medicine, The Second Xiangya Hospital, Changsha, China; ^3^ Department of General Surgery, Changhai Hospital, Navy Military Medical University, Shanghai, China; ^4^ Department of Microbiology, Immunology, and Molecular Genetics, University of Texas Long School of Medicine, UT Health Science Center, San Antonio, TX, United States

**Keywords:** soft-tissue sarcoma, senescence, molecular profiling, prediction model, immunotherapy response

## Abstract

**Background:** Soft tissue sarcoma (STS) is the malignancy that exhibits remarkable histologic diversity. The diagnosis and treatment of STS is currently challenging, resulting in a high lethality. Chronic inflammation has also been identified as a key characteristic of tumors, including sarcomas. Although senescence plays an important role in the progression of various tumors, its molecular profile remains unclear in STS.

**Methods:** We identified the senescence-related genes (SRGs) in database and depicted characteristics of genomic and transcriptomic profiling using cohort within TCGA and GEO database. In order to investigate the expression of SRGs in different cellular subtypes, single-cell RNA sequencing data was applied. The qPCR and our own sequencing data were utilized for further validation. We used unsupervised consensus clustering analysis to establish senescence-related clusters and subtypes. A senescence scoring system was established by using principal component analysis (PCA). The evaluation of clinical and molecular characteristics was conducted among distinct groups.

**Results:** These SRGs showed differences in SCNV, mutation and mRNA expression in STS tissues compared to normal tissues. Across several cancer types, certain shared features of SRGs were identified. Several SRGs closely correlated with immune cell infiltration. Four clusters related to senescence and three subtypes related to senescence, each with unique clinical and biological traits, were established. The senescence scoring system exhibited effectiveness in predicting outcomes, clinical traits, infiltrations of immune cells and immunotherapy responses.

**Conclusion:** Overall, the current study provided a comprehensive review of molecular profiling for SRGs in STS. The SRGs based clustering and scoring model could help guiding the clinical management of STS.

## Introduction

STS is rare but poses many challenges regarding to its diagnosis and treatment due to its high heterogeneity (Gamboa et al., 2020). More than 100 distinct histologic and molecular subtypes have been established for STS. According to the latest cancer statistics, the new cases of STS will reach up to 13,400 in 2023 and the deaths will be up to 5,140 (Siegel et al., 2023). Traditionally, STS has been standardly diagnosed with histology, referred as the gold standard. As molecular biology techniques continue to advance, there has been an increase in interest in the use of molecular profiling in STS, both as a diagnostic and classification tool (Italiano et al., 2016). For instance, the Ewing sarcomas are characterized by a fusion of the EWSR1 gene and FLI1 gene (85% of cases) (Ludwig et al., 2021). Several studies have explored the detailed maps of molecular and biomarkers for STS (Barretina et al., 2010; Movva et al., 2015; Italiano et al., 2017). The main characteristics of STS consist of prevalent copy number variations (CNVs), chromosomal losses, or gene fusions. While in general STS represents low mutation burden, genes including TP53, ATRX, RB1, and BRCA2 are more commonly mutated across multiple subtypes. Although there have been some novel treatment approaches like immunotherapy for STS, our current understanding of immunotherapy in STS is still at the very beginning (Martín-Broto et al., 2020).

Senescence is a cellular process that responds to various stress signals and contributes to several diseases and the aging process (Zhang et al., 2022). It is characterized by cell cycle arrest and alteration in cell morphology and physiology. Therefore, senescence serves as a protective mechanism against tumor progression (Collado and Serrano, 2010). Senescence in cancer could triggered by the activation of oncogene, such as H-RASV12 (Muñoz-Espín and Serrano, 2014), which referred as the oncogene-induced senescence (OIS). Other cancer-related signaling pathway such as activated MYC and hyperactivated WNT-β-catenin signaling also trigger senescence (Wu et al., 2007; Zhang et al., 2011). Besides, chemotherapies and radiotherapies can still force tumor cells to enter the senescence status, namely the therapy-induced senescence (Collado and Serrano, 2010; Ewald et al., 2010). The induction of senescence also leads to modifications in the tumor microenvironment, including enhanced infiltration of M1 macrophage, Th1 cells, and NK cells (Kang et al., 2011; Eggert et al., 2016). Senescence has been implicated in STS in a number of studies. Overexpression of p16 (INK4a) was identified in multiple subtypes of STS, which was associated with the induction of senescence (Knösel et al., 2014). The endogenous Ewing sarcoma gene (Ews) was involved in Ewing sarcoma progression, deletion of Ews enhances the entrance of hematopoietic stem progenitor cells into senescence (Cho et al., 2011). Therefore, it is noteworthy that targeting senescence may serve as potential therapeutics for STS. There also is now evidence from epidemiological and experimental studies that links the development of sarcoma to inflammation, which could be regarded as the inflammatory disease.

In this investigation, a systematic investigation of senescence-related genes (SRGs) in STS was presented by utilizing datasets retrieved through TCGA and GEO databases. With machine-learning algorithms, we defined the senescence-related clusters and senescence-related subtypes, as well as a senescence score model. Besides, we depicted the molecular (genome and transcriptome) and TME characteristics among different clusters or subtypes. Findings of this study could provide evidences for senescence-related biology and senescence-based therapeutic strategies for STS.

## Methods and materials

### Data preparation

The sequencing and corresponding clinical data for STS were acquired via the GEO and TCGA databases. The transcriptome data within the GTEx database for normal adipose and muscle tissues was employed as the control group. The UCSC bioinformatic pipeline (TOIL RNA-seq) was utilized to perform co-analysis of the datasets (Wang et al., 2018). The somatic mutation and CNVs were obtained through TCGA-SARC cohort as well. TARGET Pan-Cancer (PANCAN) cohort was utilized for pan-cancer analysis. We identified two bulk RNA-sequencing datasets (GSE39055 and GSE176307) and one scRNA-seq dataset through GEO database. For examining relationships among SRGs with drug responses, the study included a patient population who had undergone immunotherapeutic therapy using concomitant inhibition of both PD-1 and CTLA-4.

### Unsupervised clustering of SRGs

A total of 279 gene candidates associated with cellular senescence were acquired through the use of the CellAge database, a comprehensive cellular senescence repository (https://genomics.senescence.info/cells/). In order to perform Least Absolute Shrinkage and Selection Operator (LASSO) regression analysis, we implemented the “glmnet” package. Additionally, a tenfold cross-validation approach was employed for determining the optimal penalty regularization parameter λ. To minimize empirical error, we utilized the support vector machine recursive feature elimination (SVM-RFE) method, which follows the principle of structural risk minimization, another machine learning approach. Genomic locations of SRGs were virtualized by using “Rcircos” package. Unsupervised clustering was performed to identify clusters and subtypes associated with SRGs. MaxK = 9 and repetitions = 1,000 was set as key parameters for R package “ConsensusClusterPlus” (Wilkerson and Hayes, 2010).

### Gene set variation analysis (GSVA)

For comparing the signatures of pathways among different clusters or subtypes, GSVA analysis was performed by utilizing the R package “GSVA” (Hänzelmann et al., 2013) with predefined datasets from the MSigDB. We analyzed the data on the basis of package “limma”, while modified t-statistics were employed for visualization. We utilized the “clusterProfiler” package for performing GO annotation and set the false discovery rate (FDR) threshold as 0.05 (Yu et al., 2012). For visualizing the correlation among SRGs in STS, we utilized the “corrplot” package. For the purpose of visualizing the relationships among SRGs and prognosis, we utilized the R package “igraph”.

### Exploring the differentially expressed genes (DEGs)

We determined DEGs among the senescence-related clusters or subtypes using the R package “limma”. The p-values were adjusted based on Benjamini-Hochberg method for addressing the issue of multiple comparisons. We defined a statistically significant difference as the adjusted p-value <0.05. Specific threshold was adopted to assess the expression difference of genes.

### Quantification of immune infiltration in TME

We conducted an analysis for assessing the infiltration levels of immunocytes in STS based on single-sample gene set enrichment analysis (ssGSEA), as previously described (Bindea et al., 2013). The degree of immune infiltrations was scaled to range from 0 to 1. Relationships of TME with other biological process was evaluated by using signatures of tumor mutation burden (TMB) (Mariathasan et al., 2018). We also utilized the R package “ESTIMATE” to compute ESTIMATE scores based on gene signatures (Yoshihara et al., 2013), to assess the levels stromal and immune infiltrations within tumor. Potential signatures involved in immunotherapy response and cancer-immunity cycles were retrieved from previous research (Chen and Mellman, 2013; Qi et al., 2023). The association between senescence scores and GSVA scores was evaluated by using the R package “ggcor”.

### Development of the scoring system of senescence

For the quantification of senescence, we introduced the senescence scoring system, following the establishment of senescence-related clusters. We picked up the DEGs among different senescence-related clusters and conducted the PCA to obtain senescence score. On this setting, genes related to most set factors will display a high score and *vice versa*, as previously reported (Zhang et al., 2020; Chong et al., 2021). Equation used to calculate the senescence socre was Σ (PC1i + PC2i) (i indicates the expression levels for the selected genes based on the PCA).

### Transcriptome analysis at single-cell level

The single-cell sequencing dataset GSE131309 (Jerby-Arnon et al., 2021) was analyzed following the standard pipelines, using the Seurat package. We applied LogNormalize (scale factor = 10,000) for normalizing the gene expression. Subsequently, we used FindVariableGenes to recognize the 2,000 highly variable genes. Cell sub-population was labeled using the annotation methods described in previous publication (Jerby-Arnon et al., 2021).

### Predicting chemotherapy sensitivity

The Genomics of Drug Sensitivity in Cancer (GDSC) is the publicly available source that contains molecular features of cancers predicting response to anti-cancer drugs. The database records thousands of tumor cell lines and 518 compounds. For determining corresponding IC50 and drug sensitivity score, we used the “pRRophetic” and “oncoPredict” (Iorio et al., 2016; Maeser et al., 2021).

### Cell lines and clinical samples

Four STS cell lines were used in this study, including SW-872, hSS-005R, SW-982 and HSF. SW-872, SW-982 and HSF were obtained from ATCC while hSS-005R was established by our laboratory. In the 37°C environment with 5% CO_2_, the cell lines were grown in Dulbecco’s modified Eagle medium (DMEM) supplemented with 10% fetal bovine serum (FBS).

We collected and further sequenced four pairs of STS samples as well as their corresponding normal tissues using Oxford Nanopore Technologies (Oxford, United Kingdom). The sequencing matrix is available under accession number GSE198568. We further verified corresponding expression for SRGs in this dataset.

### Real time quantitative PCR (RT-qPCR)

We used the RNA Express Total RNA Kit (M050, NCM Biotech, China) to extract total RNA from the cells. Following the removal of genomic DNA, we used The RevertAid First Strand cDNA Synthesis kit (K1622, Thermo Fisher Scientific, United States) for synthesizing cDNA from the total RNA. Detailed steps were similar to previous study (Qi et al., 2022; Qi et al., 2023). As shown in Supplementary Table S1, this study utilized the following primers.

### Cell transfection

The Hanbio (Shanghai, China) designed and synthesized the siRNA for E2F1 knockdown, as well as the corresponding negative control (siNC). The cells were seeded into a 6-well plate with the concentration of 1.5 × 10^5^ cells per well. The 50 nmol of siRNA or siNC was further transfected into the cells using 5 μL of Lipofectamine 2000 reagent. Following a 6-h transfection period, fresh medium was further added to the culture medium. The siRNA sequences were listed in Supplementary Table S2. We collected or analyzed the cells 48 h after transfection and used them for subsequent experiments.

### Cell proliferation assay

We plated cells into a 96-well plate with the concentration of 2000 cells per well and cultured overnight. The cell counting kit-8 (CCK-8) was utilized for testing the cells that underwent transfection after being cultured after the indicated periods of time. For each well, we added 100 μL of 10% CCK-8 solution was added, followed by 1.5 h of incubation. The absorbance at 450 nm for each well was detected by using the Tecan Spark^®^ multimode microplate reader.

### Clone formation assay

After transfection with siRNA, cells were seeded in 6-well plates with the concentration of 1,000 cells per well and subsequently cultivated for the indicated durations. At the end of experiment, cellular clones were fixed utilizing 4% paraformaldehyde (PFA) and subsequently subjected to staining by using 0.2% crystal violet solution with an additional period of 15 min.

### Wound healing assay

In order to assess the migratory capabilities of the cells, we utilized the wound healing assay. Cells were seeded into 6-well plates with the concentration of 4 × 10^5^ cells per well. Upon achieving approximately 80% cellular confluence, we created a scratch employing the 100 μL pipette tip. Subsequently, cells were cultivated in DMEM with 2% FBS. The area covered by the migrated cells was then measured after indicated duration, by the light microscope.

### Statistical analysis

This study employed the R statistical analysis software (version 4.1.0) as part of its methodological approach. Spearman’s correlation test was employed for determining the associations among the studied SRGs. Parametric comparisons were calculated by utilizing Student’s t-tests while nonparametric comparisons were determined through the Wilcoxon signed-rank test. Comparisons across multiple groups were ascertained through the implementation of one-way ANOVA or the Kruskal-Wallis test. Patient survival outcomes were comparatively analyzed utilizing the Log-rank test. Utilizing both univariate and multivariate Cox regression analyses, the predictive determinants were identified. In order to establish the threshold value for the senescence score, the “surv_cutpoint” function within the “survminer” package was employed. After determining optimal senescence score cutoff value, The patients were stratified into two groups characterized by high and low senescence levels. Following this, Chi-square tests or Fisher’s exact tests were applied to compare their clinical attributes.

## Results

### Selection of candidate SRGs

**FIGURE 1:**
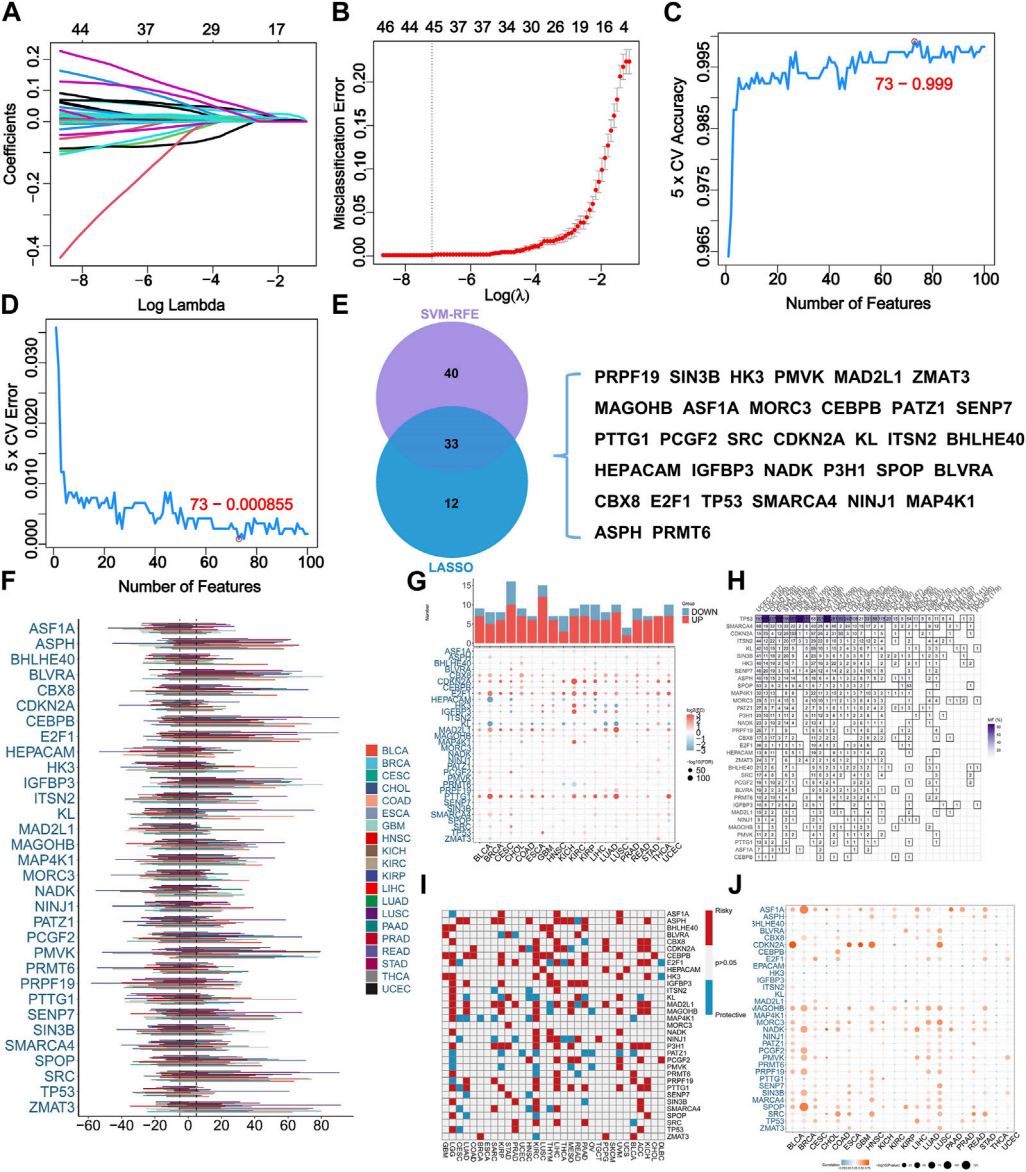
Identification of senescence-related genes (SRGs) based on machine learning algorithms and pan-cancer analysis. **(A)** LASSO coefficient profiles of the 279 SRGs. **(B)** LASSO cross‐validation for selecting optimal tuning parameter (λ). **(C)** The error rate of SVM-RFE modeling across varying feature counts. **(D)** The accuracy rate of SVM-RFE modeling across varying feature counts. **(E)** The overlapping of selected SRGs based on two algorithms. **(F)** The somatic copy number variance (SCNV) of SRGs across various cancers. **(G)** Comparative analysis for expressing levels of SRGs in tumor tissues with corresponding normal tissues across various cancers. **(H)** The frequency of SRG mutations across various cancers. **(I)** The prognostic roles of SRGs across various cancers. The use of red and blue colors corresponds to factors associated with poor and favorable prognosis, respectively. **(J)** The correlation analysis for the expressing levels of SRGs and SCNV across various cancers.

Collectively, 279 SRGs were identified based on the database. Two machine learning-based algorithms, namely LASSO regression analysis and SVM-RFE, were employed to analyze the input data, in which 45 and 73 SRGs were identified respectively (Figures 1A–D). 33 SRGs overlapped by result of two algorithms were used for downstream analysis (Figure 1E).

### Pan-cancer analysis of SRGs

For shedding lighting on illumination of the characteristics of SRGs in STS. We explored the SCNV in pan-cancer level. Results indicated the SCNV gain in ASPH, BLVRA, CEBPB, E2F1, IGFBP3, PMVK, SENP7, SPOP, SRC, and ZMAT3 (Figure 1F). Interestingly, E2F1 was highly expressed in most cancer types (Figure 1G). TP53 and SMARCA4 showed high frequency of mutation among the 33 analyzed SRGs (Figure 1H). ASPH, CDKN2A, E2F1, P3H1, and PTTG1 were identified as prognostic indicators of unfavorable outcomes within multiple cancer types (Figure 1I). Additionally, the CNV condition and expression level of ASF1A, CDKN2A, MAGOHB, NADK, SPOP, and ARC were remarkably correlated in most cancer types (Figure 1J).

### Genome and transcriptome characteristics of SRGs

**FIGURE 2:**
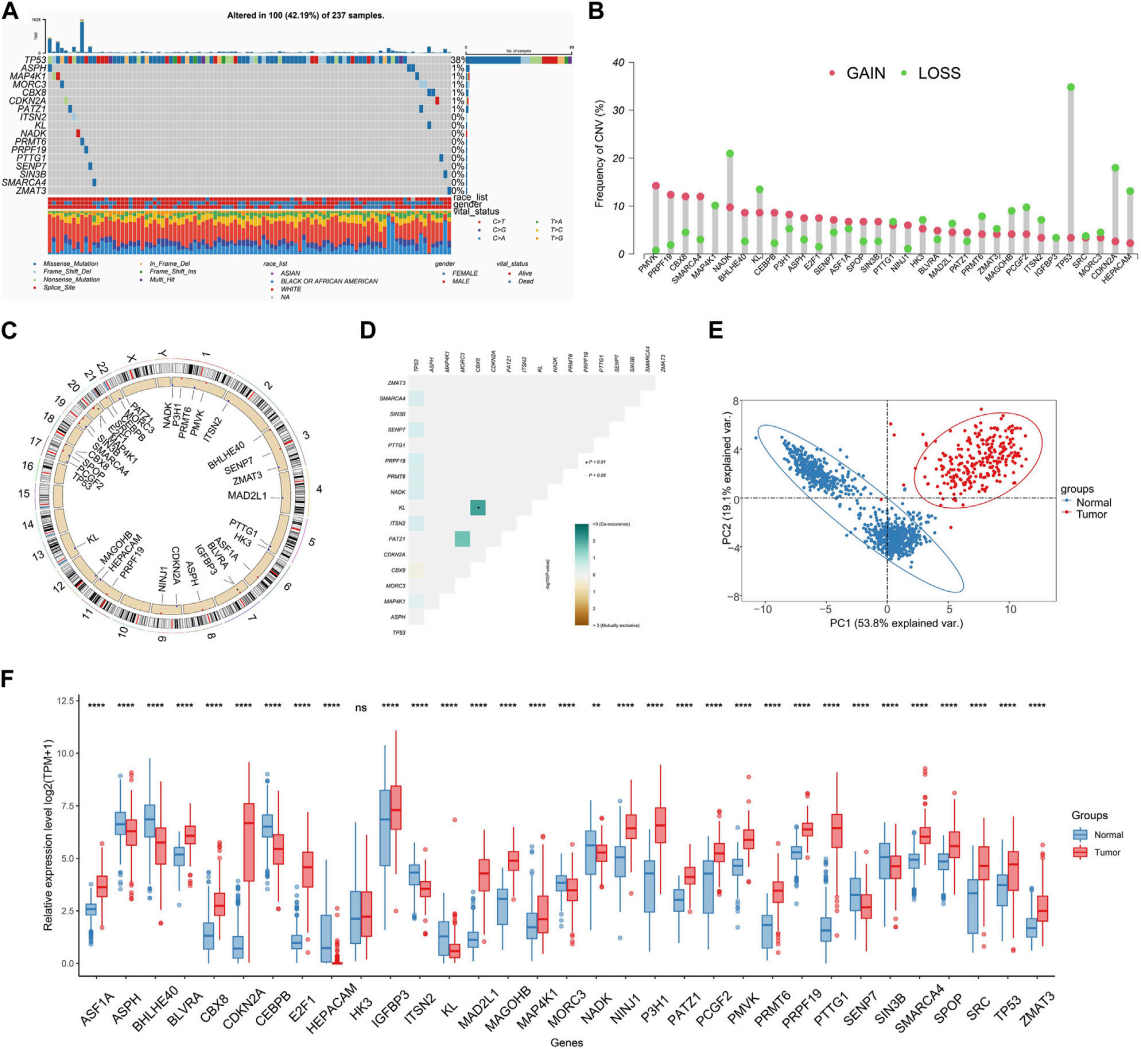
Characteristics of SRGs in genome and transcriptome. **(A)** The frequency of SRG mutations (Top 8) within TCGA-SARC cohort. **(B)** The CNV gain and loss of SRGs in TCGA-SARC cohort. **(C)** The chromosomal localization of SRGs within humans. **(D)** Analysis of co-occurring and mutually exclusive mutations in SRGs. **(E)** Principal component analysis (PCA) was conducted for differentiating STS from normal tissues on the basis of the expression patterns of SRGs. **(F)** Comparative analysis for expressing levels of SRGs in STS and normal tissues by utilizing the TCGA-GTEx database.

We identified mutations related to SRGs in TCGA-SARC cohort. Results demonstrated that a large number of samples present SRGs-associated mutations (altered in 100 of 237 samples) (Figure 2A). The frequency of CNV of SRGs is displayed in Figure 2B. PMVK showing the highest CNV gain while TP53 acquiring the highest CNV loss. Most SRGs locate at chromosome 1 and 17 (Figure 2C). Interaction among somatic mutations of SRGs was measured by somatic interaction function. KL and CBX8 exhibited high co-occurrence with each other (*p* < 0.05) (Figure 2D). We found that expressing levels of 33 SRGs were effective in discriminating between tumors and normal tissues (Figures 2E, F).

**FIGURE 3:**
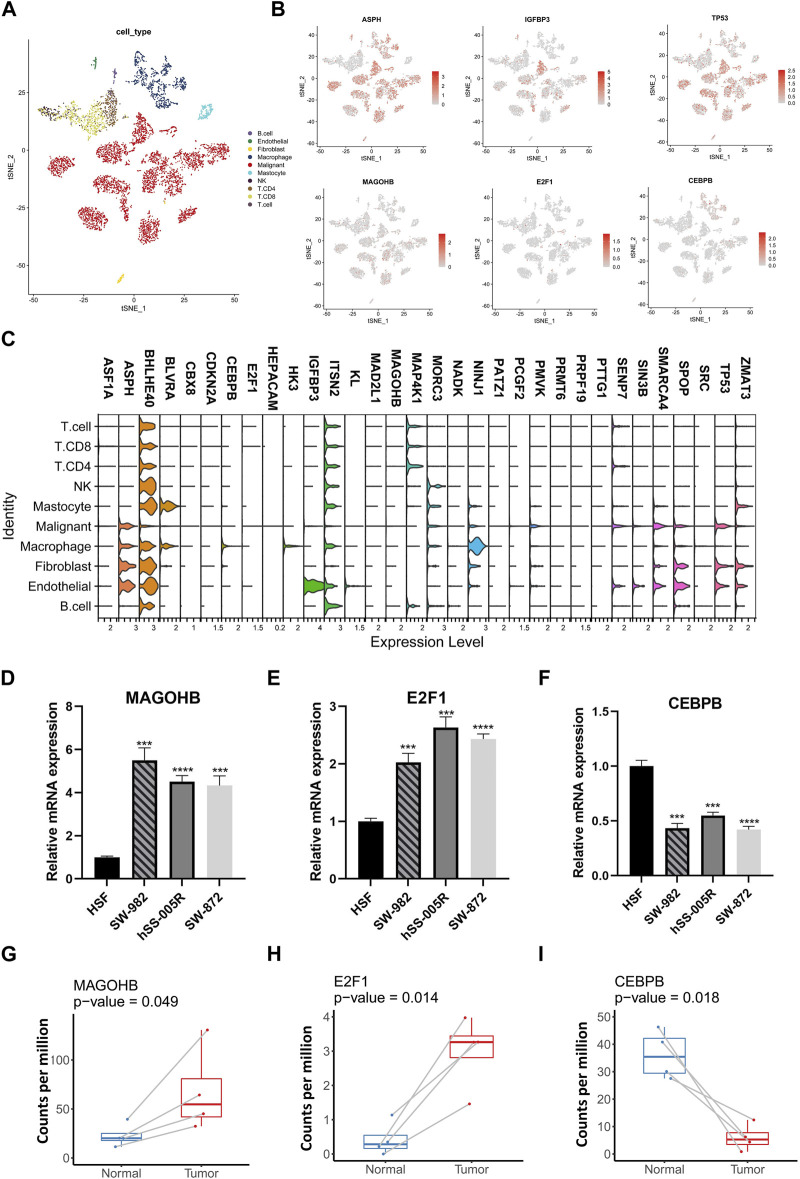
The single-cell resolution expressing patterns of SRGs and validation in cell lines. **(A)** The t-distributed stochastic neighbor embedding (t-SNE) of different cellular clusters in GSE131309. **(B)** The expression patterns of specific SRGs across diverse cellular types. **(C)** The violin plots illustrating expressing levels of specific SRGs. **(D–G)** The expressing levels of specific SRGs in STS cell lines were validate by using qPCR. **(H,I)** Comparative analysis for SRGs among STS and matched adjacent normal tissues by using own sequencing data.

Expression pattern of SRGs was further analyzed at single-cell level using data from GSE131309 (Figure 3A). We noticed that ASPH, BHLHE40 and ITSN2 showed wide expression in different cell types while IGFBP3, CDKN2A, HK3 and MAP4K1 expressed in specific cell types (Figures 3B, C; Supplementary Figure S1). Consistently, expression of MAGOHB and E2F1 were upregulated while CEBPB was downregulated in STS cell lines and our clinical samples (Figures 3D–I).

**FIGURE 4:**
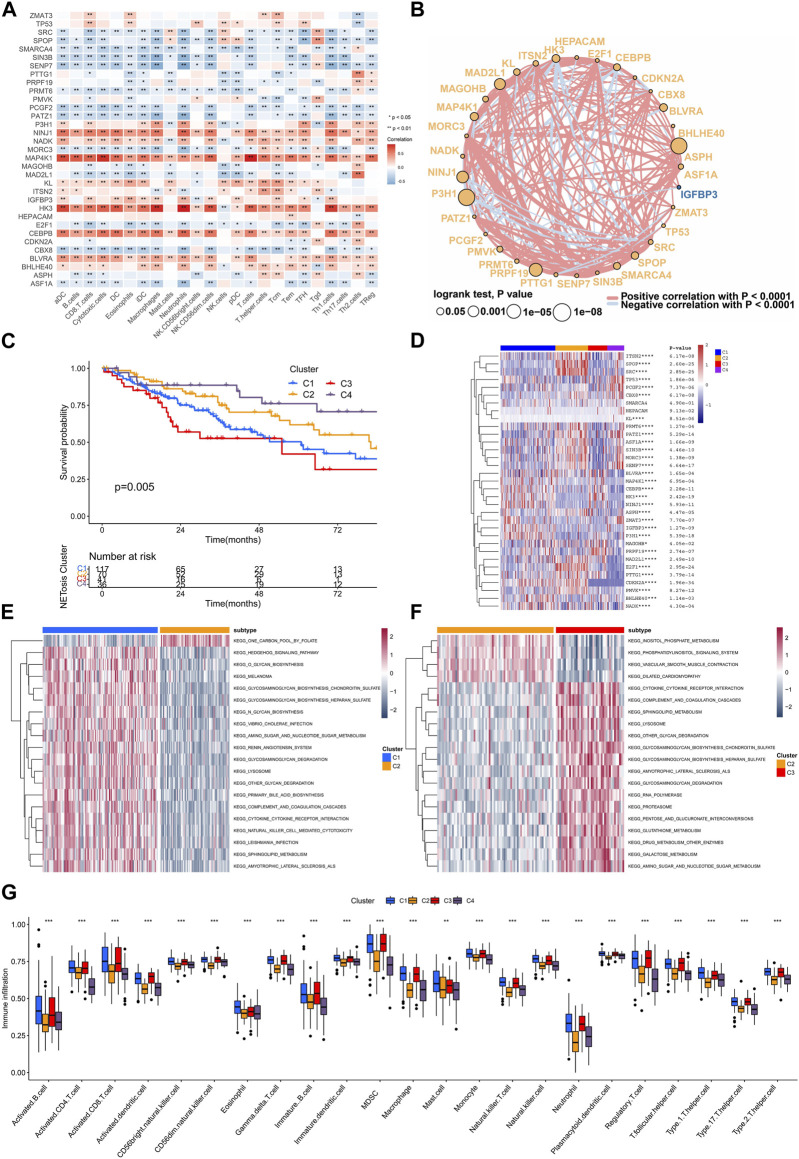
Interaction between SRGs and identification of clusters related to senescence. **(A)** The association of expressing levels of SRGs with the immune cells signatures. **(B)** The interaction networks of SRGs within TCGA-SARC cohort. **(C)** Kaplan-Meier analysis for different senescence-related clusters. **(D–F)** The gene set variation analysis (GSVA) showing different enriched pathways among various senescence-related clusters. **(G)** The infiltrations of various immune cells among different senescence-related clusters.

TME is a critical mediator of within tumor characteristics, as it involves interactions among immune cells and cancer cells and can influence their fate. We conducted an analysis to examine associations among immune signatures and expression of genetic factors. The results of the analysis showed that NINJ1, MAP4K1, HK3, and CEBPB exhibited a positive correlation with the majority of immune cell types (Figure 4A). A comprehensive correlation analysis of SRGs was also conducted, which showed the wide positive association between most SRGs. However, N3H1, NINJ1, HK3, and CEBPB were negatively correlated with most SRGs (Figure 4B).

### Senescence-related clusters and interactions

Consensus unsupervised clustering was performed on TCGA-SARC cohorts, which resulted in the division of patients into distinct groups according to the expressing levels of 33 SRGs (Supplementary Figure S2A–F). Consequently, four clusters were identified according to the optimal clustering number, containing 117 cases within cluster C1, 70 cases within cluster C2, 41 cases within cluster C3 and 36 cases within cluster C4. They showed difference in survival rate, in which the cluster C4 displaying best prognosis (Figure 4C). The four clusters had distinct expression patterns of 33 SRGs (Figure 4D). Further, we applied the GSVA analysis to compare the clusters enriched in different clusters. As a result, cluster C2 was found to be exhibit negative enrichment within pathways related to cytokine-cytokine receptor interaction, complement and coagulation cascades, lysosome, sphingolipid metabolism, and amino sugar and nucleotide sugar metabolism (Figures 4E, F; Supplementary Figure S2G–J). Interestingly, cluster C2 showed significant downregulated adaptive immune cell infiltration (Figure 4G).

### Identification of senescence-related subtypes

**FIGURE 5:**
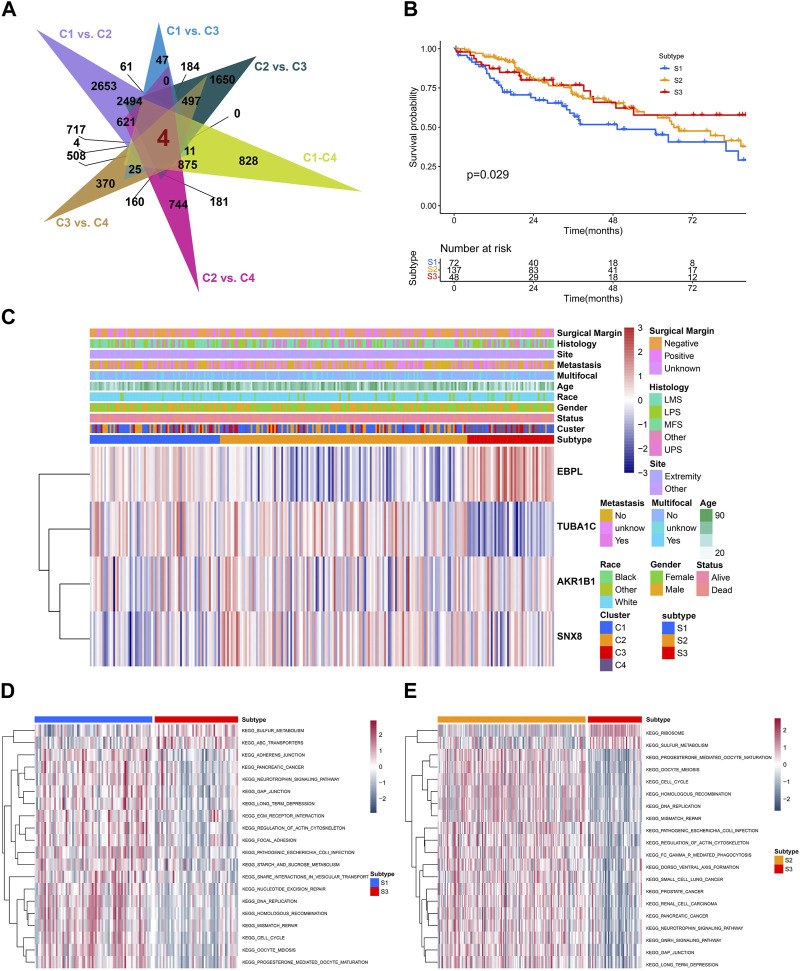
The biological and prognostic role of senescence-related subtypes. **(A)** The overlapping of differentially-expression genes (DEGs) with senescence-related clusters. **(B)** The Kaplan-Meier analysis of different senescence-related subtypes. **(C)** The heatmap showing the clustering of senescence-related subtypes based on overlapped DEGs. **(D,E)** The GSVA showing different enriched pathways among different senescence-related subtypes.

To further reveal the characteristics of different clusters, we calculated the senescence-related DEGs, only four overlapping DGEs were identified among different clusters (Figure 5A). Unsupervised consensus clustering was carried out by utilizing DEGs, for identifying subtypes associated with senescence (Supplementary Figure S3A–F). Consequently, the study identified three distinct subtypes, denoted as S1, S2, and S3, containing 72, 137, 48 patients respectively. They showed significant difference in outcomes and heterogeneity in clinical features (Figures 5B, C). Moreover, the GSVA analysis indicated that subtype S3 was positively enriched in sugar metabolism while negatively enriched in DNA repair pathways such as the DNA replication, mismatch repair, cell cycle, homologous recombination (Figure 5D; Supplementary Figure S3G).

### Development and verification of senescence score

**FIGURE 6:**
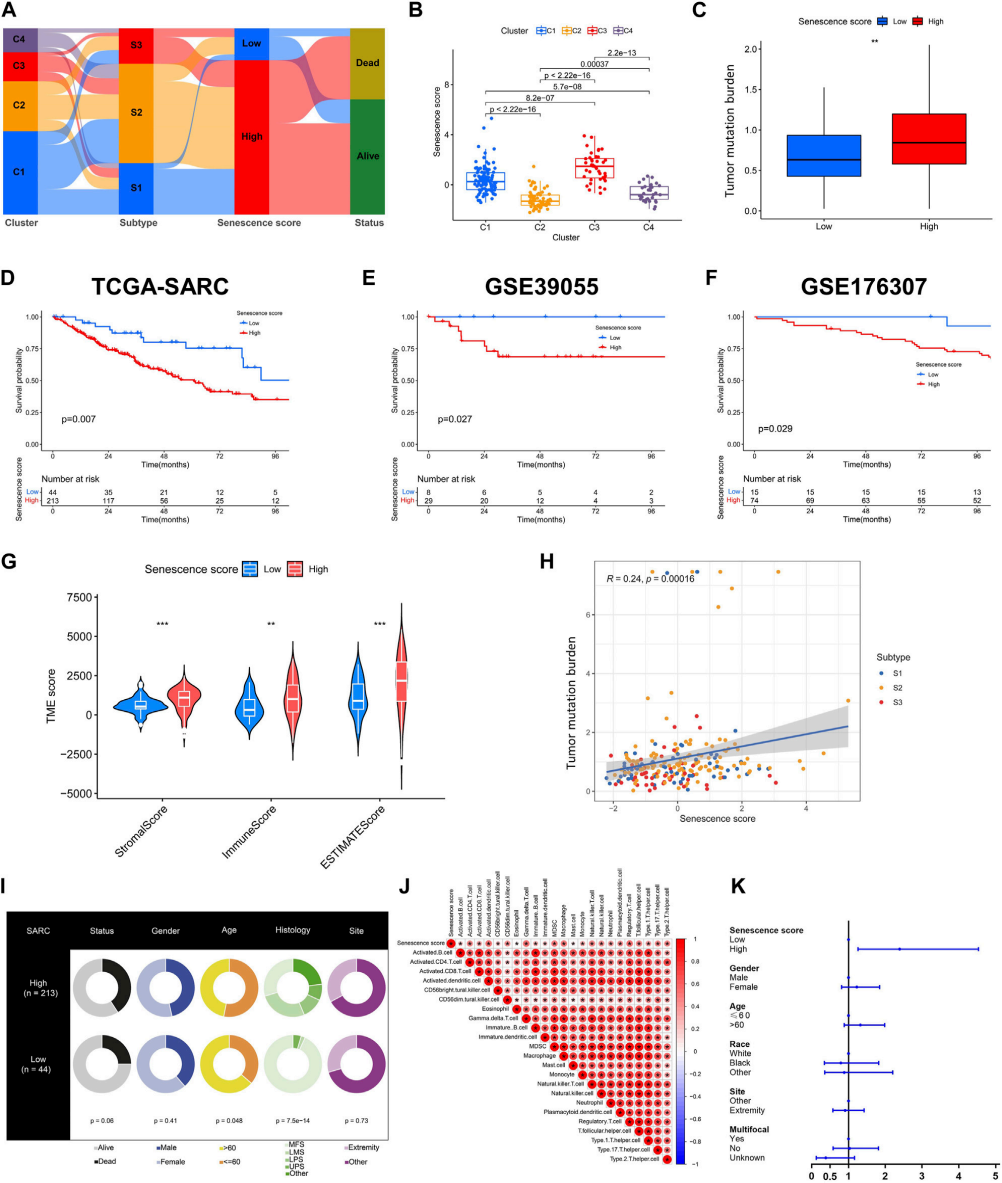
Construction and validation of senescence score. **(A)** The alluvial diagram illustrating the relationships within senescence-related clusters, subtypes, scores and survival status. **(B)** Box plots showing senescence scores of different senescence-related clusters. **(C)** Box plots showing tumor mutation burden (TMB) within low and high senescence scores. **(D–F)** Kaplan-Meier analysis of validation for the prognosis role of senescence scores in TCGASARC, GSE39055 and GSE176307 cohorts. **(G)** The scores of tumor microenvironment (TME) between low and high senescence scores based on ESTIMATE algorithm. **(H)** The correlation analysis of senescence scores, senescence-related subtypes and TMB. **(I)** The table of pie plots showing the clinical characteristics within low and high senescence scoring cohorts. **(J)** Correlational analysis between senescence scores with immune cell signatures. **(K)** The forest plot showing the multivariate Cox regression analysis of senescence scoring model and clinical characteristics.

To establish an individualized predictive model for senescence, we subsequently calculated the senescence score based on senescence related DEGs (Figure 6A). As expected, senescence-related clusters showed significant differences in senescence score (Figure 6B). A marked disparity in tumor mutation burden was observed between the high and low senescence scoring cohorts (Figure 6C). Notably, individual exhibiting high senescence scores exhibited unfavorable prognosis, as validated in three datasets (TCGA-SARC, GSE39055, GSE176307) (Figures 6D–F). The TME scores, encompassing stromal, immune, and ESTIMATE scores, demonstrated marked variation between the high and low senescence score cohorts (Figure 6G). Interestingly, we found a positive correlation among the senescence scoring and tumor mutation burden (Figure 6H). Distinct clinical characteristics were observed between the high and low senescence score groups, including age (*p* = 0.048) and histology (*p* < 0.001) (Figure 6I). Moreover, a positive correlation was discovered between senescence score and several immune cell subtypes, including activated CD4^+^ T cell, γδT cell, MDSC and macrophage (Figure 6J). In addition, the multivariate Cox regression analysis demonstrated that the senescence scoring exhibited significant prognostic value as a unfavorable prognostic indicator within STS (Figure 6K; Supplementary Figure S4A–I).

### Biological features associated with senescence scores

**FIGURE 7:**
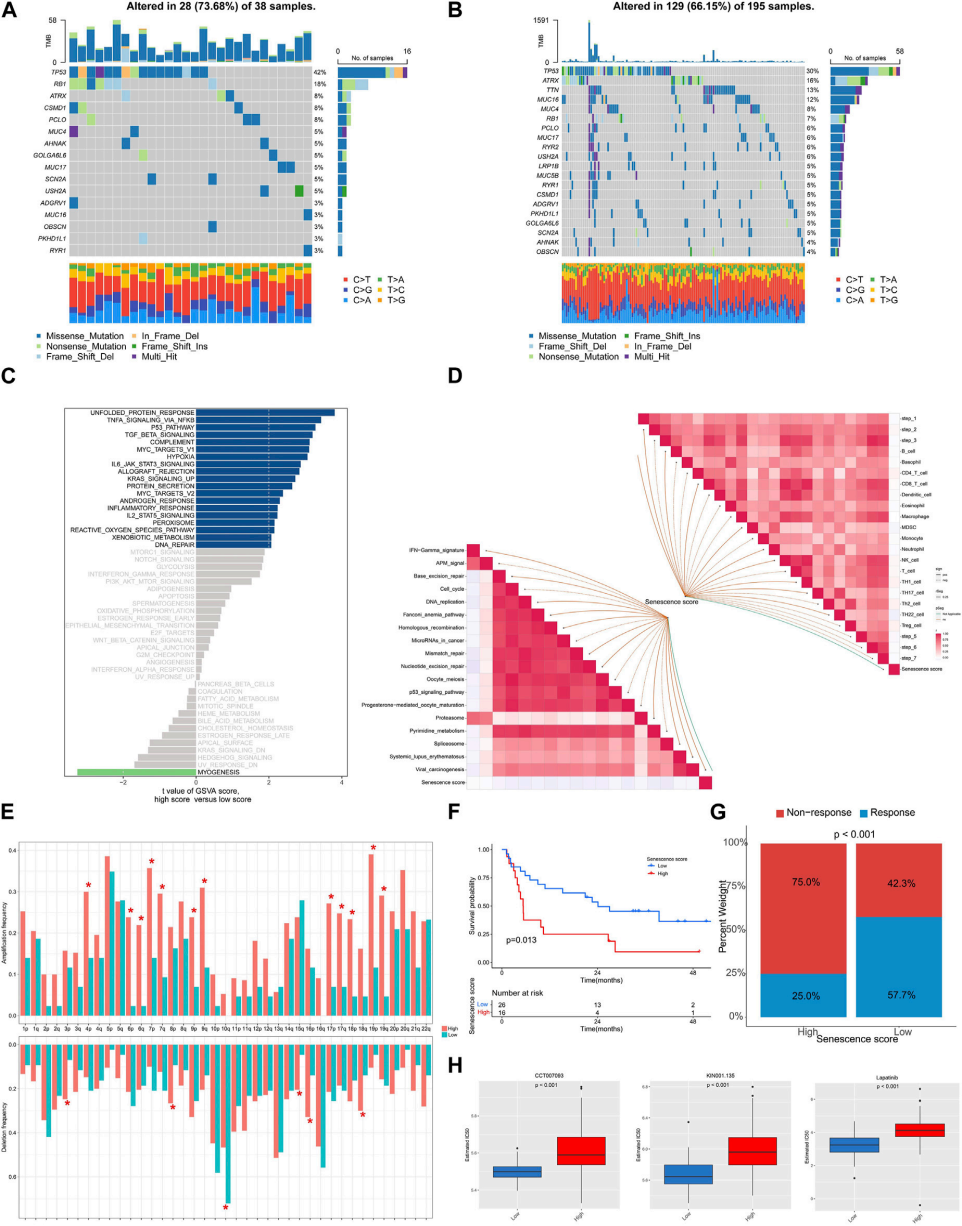
The genomic and transcriptional characteristics of senescence scoring model in TCGA-SARC cohort. **(A,B)** The top mutated genes among low and high senescence scoring cohorts. **(C)** Bar plots showing the GSVA for hallmark pathways between low and high senescence scoring cohorts. **(D)** The association between senescence score and predicted pathways for immunotherapy and cancer immunity cycles. **(E)** The frequencies of amplification and deletion at the arm level among the low and high senescence scoring cohorts. **(F)** Kaplan-Meier analysis was utilized to compare the low and high senescence score groups in the immunotherapy-treated cohort. **(G)** The proportion of clinical response among low and high senescence score groups treated with immunotherapy. **(H)** The drugs with significantly different estimated IC50 among low and high senescence scoring cohorts.

We subsequently depicted the differences of genomic and transcriptomic profiles between high and low senescence scores groups. As illustrated, a lower mutation frequency was noticed within the cohort exhibiting higher senescence score, wherein alterations within 129 (66.15%) of 195 individuals (Figures 7A, B). Notably, the arm level amplification was more prevalent in high senescence score cohort while depletion was more common in low senescence score group (Figure 7E). GSVA analysis indicated the positive enrichment of unfolded protein response, TNF signaling via NFKB, p53 pathway, TGFβ pathway and MYC targets in high senescence score group (Figure 7C), but the pathways of myogenesis was negatively enriched (Figure 7C). Furthermore, we analyzed senescence score’s association with immunotherapy-related pathway and cancer immunity cycles. Senescence score was positively correlated with several subtypes of immune cells including CD8^+^ T cell, dendritic cell, macrophage and NK cell (Figure 7D). Meanwhile, a positive correlation between senescence score and several immunotherapy-related pathways was identified, including IFN-γ signature, APM signal and Proteasome (Figure 7D).

To examine the association among senescence scoring system and response to immunotherapy, we analyzed a cohort of patients who had received immunotherapy. The findings revealed an unfavorable prognosis among patients exhibiting elevated senescence scores (*p* = 0.013) and a higher non-response rate when treated with immunotherapy (*p* < 0.001) (Figures 7F, G). We further screened the GDSC database and noticed a statistically significant increase in IC50 values for CCT007093, KIN001.135, and Lapatinib among the group with high senescence scores (Figure 7H).

### Effects of E2F1 on STS cells

**FIGURE 8:**
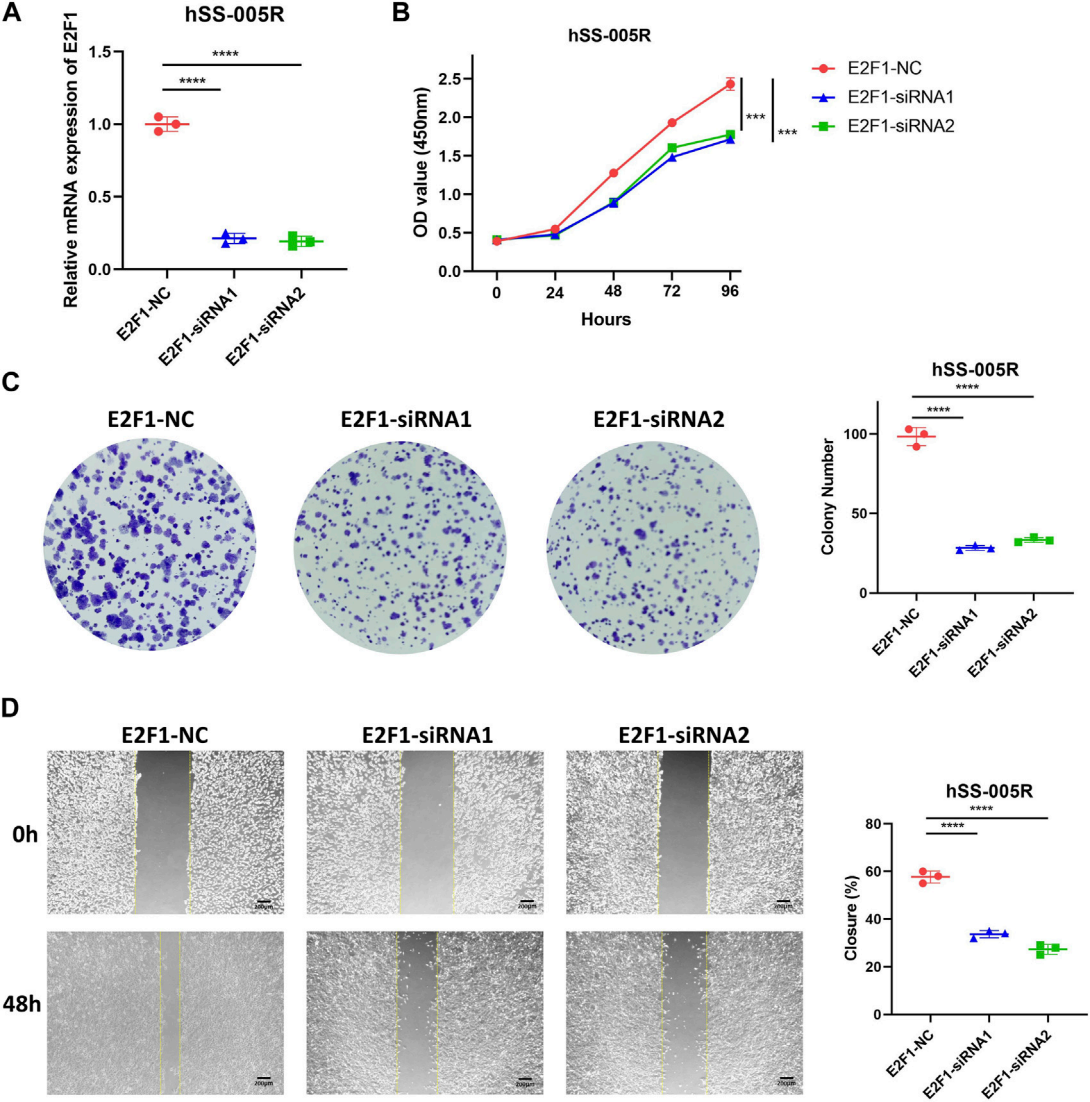
E2F1 promotes the malignant biological traits of STS cell lines. **(A)** Knockdown efficiency for E2F1 in hSS-005R cell lines. **(B)** CCK-8 assay between hSS-005R cell lines treated with E2F1 siRNA versus those treated with control siRNA. **(C)** Clone formation between hSS-005R cell lines treated with E2F1 siRNA versus those treated with control siRNA. **(D)** Scratch assay between hSS-005R cell lines treated with E2F1 siRNA versus those treated with control siRNA.

As aforementioned, E2F1 showed high expression in STS, thus we further explored its role in STS. Our findings revealed that demonstrated that the downregulation of E2F1 significantly suppressed STS proliferation, as evidenced by CCK-8 assay and clone formation (Figures 8A–C). Moreover, scratch assay demonstrated that suppression of E2F1 significantly impeded the migration of STS cell lines (Figure 8D).

## Discussion

While personalized cancer management shows advantages, it is evident that the molecular profiling is indispensable in STS classification and treatment decision (Conrad et al., 2017). With high heterogeneity, diagnosis and classification of STS remain challenging. To cope with this limitation, accumulating studies have attempted to discriminate the subgroups of STS by genomic or transcriptomic alterations. Unlike other epithelial malignancies, STS is characterized by high prevalence of CNV but low mutational burden (Abeshouse et al., 2017). The varied genomic and regulomic profiles can assist in defining molecular subtypes that are associated with patient prognosis. Approximately 31.7% of patients with sarcoma exhibited detectable changes in their genome, which included rearrangements of a significant proportion of kinase genes (Gounder et al., 2022). The DNA methylation pattern is also efficient in classification of sarcoma (Koelsche et al., 2021). Therefore, the molecular profiling is a useful tool in STS diagnosis and classification. Senescence is known to play a key role in the initiation and advancement of neoplastic growths, as it can induce growth arrest of tumor cells and against tumor cell proliferation (Calcinotto et al., 2019). Studies have shown that the overexpression of certain oncogenes or drug therapy can induce cell senescence in tumors (Prasanna et al., 2021). Therefore, the expression characteristics of senescence-related genes can also serve as molecular basis for tumor diagnosis and treatment. This study involved an in-depth examination for genes associated with cellular senescence and the development of the predictive model based on senescence for STS.

To depict common characteristics of SRGs in different cancer types, we first analyzed SRGs at a pan-cancer level. SCNV gain was identified in several genes such as E2F1, ASPH, BLVRA, and CEBPB across multiple cancers. Previous studies also uncovered high burden of E2F1 CNVs which drive tumor susceptibility in many caner types (Nelson et al., 2006; Rocca et al., 2017; Rocca et al., 2019; Rocca et al., 2021). Notably, E2F1 and ASPH were associated with poor outcomes of multiple cancers, consistent with previous studies (Lin et al., 2019; Holtzman et al., 2021; Mandigo et al., 2021; Jing et al., 2022). Next, we explored mutations of SRGs in TCGA-SARC cohort. In spite of facts that STS harbors low mutation burden, the mutation rate for SRGs was relatively high (altered in 100 of 237 samples). As similar to pan-cancer, PMVK also showed high CNV gain in STS. Differentially expression of most SRGs were found between STS tissues and normal tissues. Using STS cell lines and our clinical STS samples, we further confirmed the significantly different expression of MAGOHB, E2F1 and CEBPB in STS. At single cell resolution, we found that several SRGs such as ASPH, BHLHE40 and ITSN2 were widely expressed in multiple cell types while genes including IGFBP3, CDKN2A, HK3 and MAP4K1 expressed in specific cell types. Such expression profiles of SRGs may help uncovering the cell type-specific therapeutics.

Subsequently, we build the clustering system for STS on the basis of 33 SRGs. Four clusters were established (C1, C2, C3, C4), in which cluster C2 exhibited negative enrichment in immune-related pathways such as cytokine-cytokine receptor interaction and complement, as well as metabolism-related pathways inclduing sphingolipid metabolism and amino sugar and nucleotide sugar metabolism. Consistently, immune infiltration analysis also indicated the decreased immune cell infiltration in cluster C2. In order to classify patients based on SRGs, we calculated DEGs among different clusters and utilized unsupervised consensus clustering for identifying senescence-related subtypes. Further identification of three subtypes (S1, S2, S3) was accomplished. Our observations revealed that subtype S3 was positively enriched in sugar metabolism pathway but negatively enrich in several DNA repair pathways. Sugar metabolism activation or reprograming is an important driver of cancer progression (Hay, 2016; Abdel-Wahab et al., 2019). Targeting sugar metabolism in STS belonging to S3 subtype may be effective. Unsupervised clustering has been reported as an efficient tool for clustering distinct subtypes in specific cancer types (Chu et al., 2020; Cao et al., 2021). Further study could focus on comprehensively analyzing the characteristics of metabolism to uncover novel therapeutic targets for STS patients with specific sugar metabolism niche.

Furthermore, we established a senescence scoring system for quantification of senescence molecular profiles in individual patients. We observed significant differences of tumor mutation burden, patient outcome, TME and several clinical characteristics between high and low senescence score cohort. The high senescence score group was positively enriched in TNF signaling via NFKB, p53 pathway and TGFβ pathway. These pathways were all associated with cancer progression, as reported in other cancers (Joerger and Fersht, 2016; Colak and Ten Dijke, 2017; Schlein and Thamm, 2022). Not surprisingly, patients with high senescence score displayed poor prognosis. Additionally, high senescence score also indicated poor response to immunotherapy. Although this senescence scoring system is effective, the high heterogeneity among patients may limit its application, thus larger scale cohort is required to improve the current model.

More specifically, E2F1 was observed to be highly expressed in STS and closely related to CNV events and prognostic outcome. We further explored its role in STS by experiments. The findings of the study indicated that the suppression of E2F1 resulted in a notable reduction within the growth, proliferation, and migratory capacity of STS cells. Similarly, high expression of E2F1 promotes the occurrence and progression in other cancers (Farra et al., 2019; Jing et al., 2022; Lin et al., 2022). Therefore, E2F1 may act as a potential therapeutic senescence-related target for STS. However, the oncogenic role of E2F1 in STS was not well explored in the current study, while our findings still valuable insights of E2F1. Subsequent investigations could further analyze the underlying mechanism of E2F1’s oncogenic function in STS. Especially, this regulatory role may be attributed to the modulation of cell cycle progression, given that E2F1 is a renowned transcription factor that governs cell cycle genes (Ertosun et al., 2016). Besides, E2F1 could also participate in other oncogenic pathways such as DNA damage response or apoptosis (Biswas and Johnson, 2012; Pützer and Engelmann, 2013).

## Conclusion

Taken together, we comprehensively analyzed the senescence pattern and SRGs in STS, part of them were confirmed by experiments. For the first time, we revealed the profiling of SRGs in STS and established the senescence-related clusters and subtypes. To broaden the application of our results, we build a senescence scoring system that enables personalized evaluation of both prognosis and immune response in STS patients. These findings could deepen our understanding of senescence in STS and help uncovering novel senescence-based therapeutics.

## Data Availability

Publicly available datasets were analyzed in this study. This data can be found here: UCSC Xena (https://xena.ucsc.edu/) and GEO database (https://www.ncbi.nlm.nih.gov/geo/) with accession Nos.GSE39055, GSE176307, GSE131309, GSE198568.
