# Supplementary figures and images for: Integration analysis of senescence-related genes to predict prognosis and immunotherapy response in soft-tissue sarcoma: evidence based on machine learning and experiments
Source: Front Pharmacol. 2023 Jul 11;14:1229233. doi: 10.3389/fphar.2023.1229233 (PMC10367114; doi:10.3389/fphar.2023.1229233)

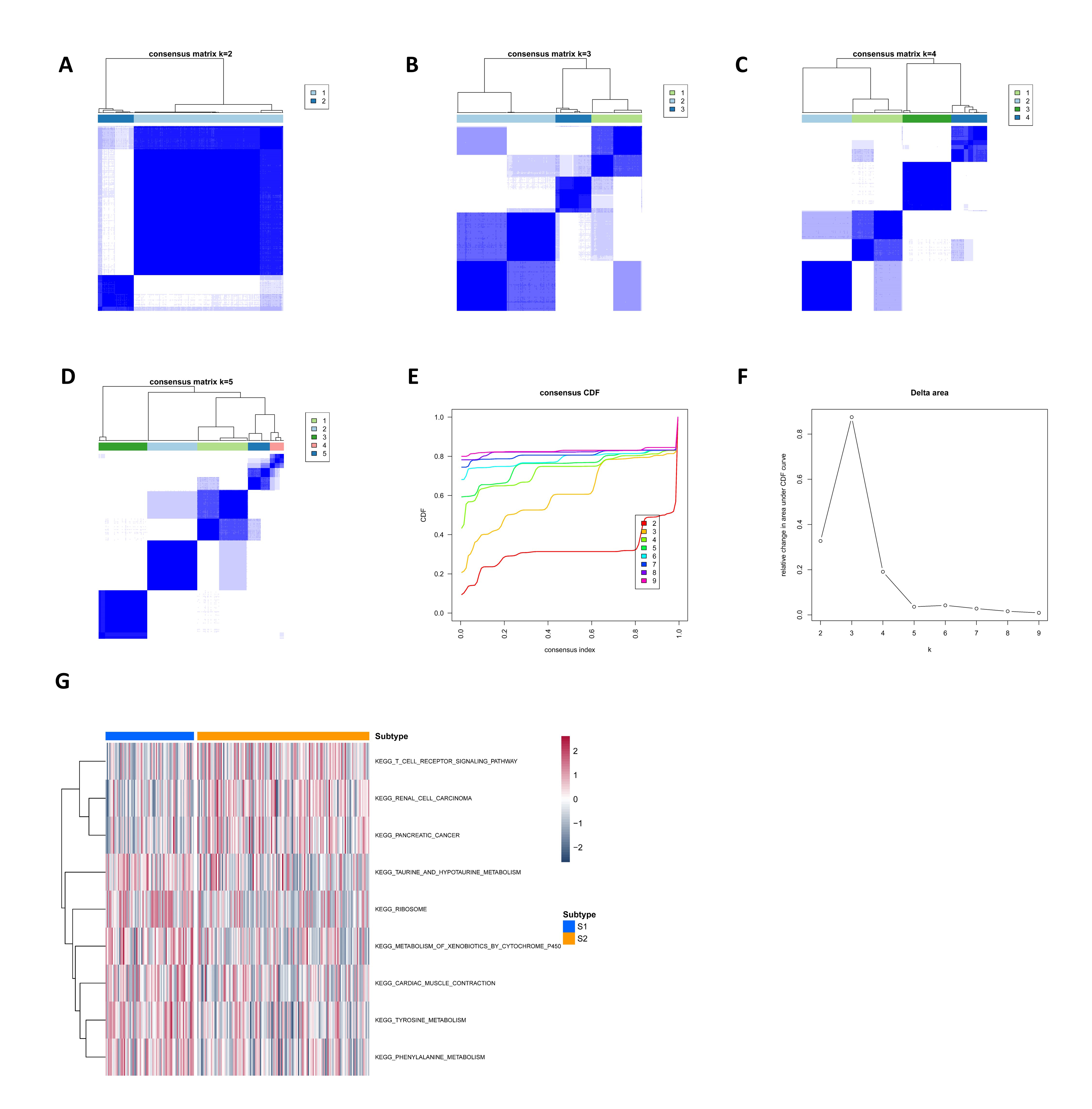

Supplement: Supplementary file 1 [file Image3.JPEG]

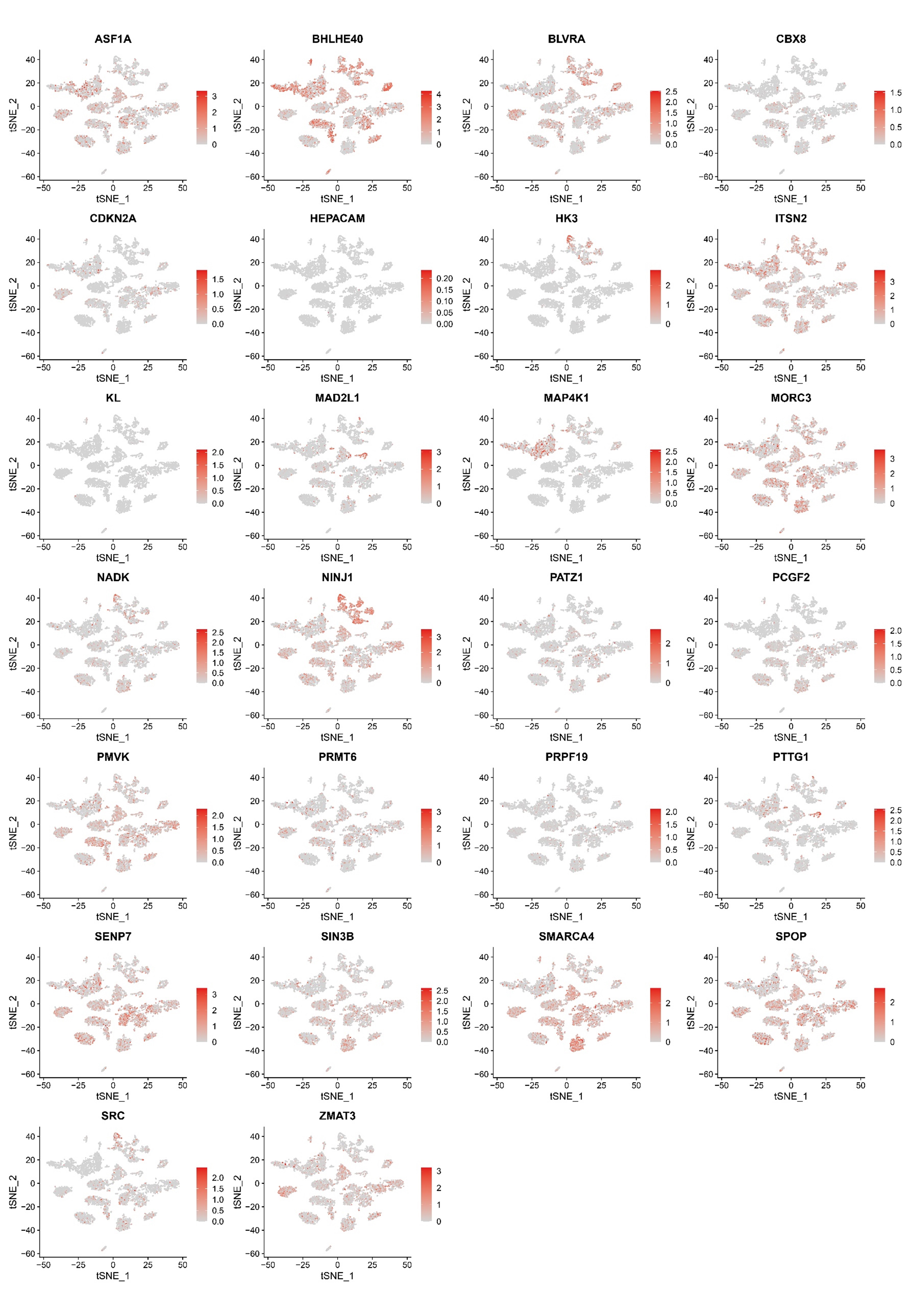

Supplement: Supplementary file 2 [file Image1.JPEG]

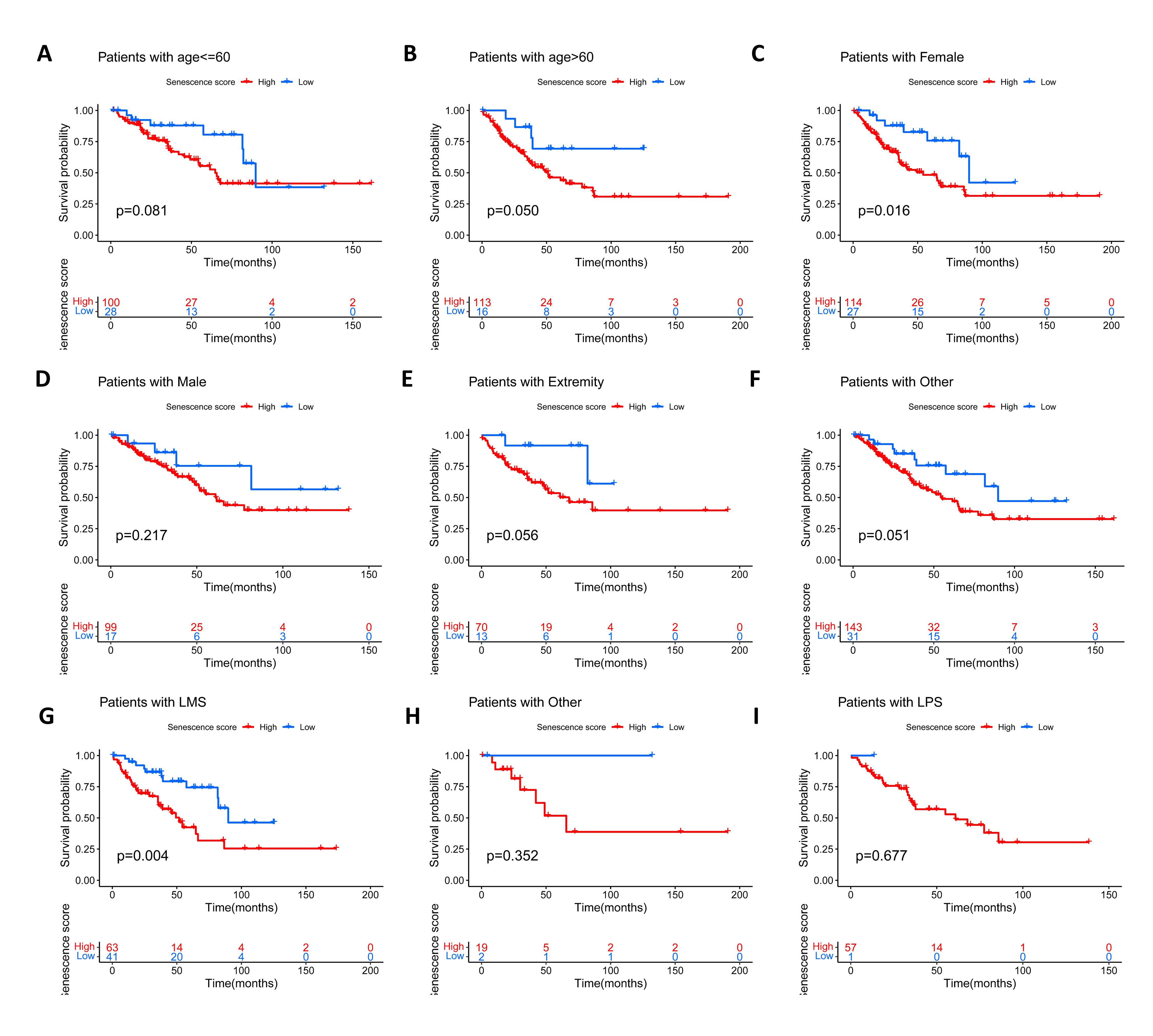

Supplement: Supplementary file 3 [file Image4.JPEG]

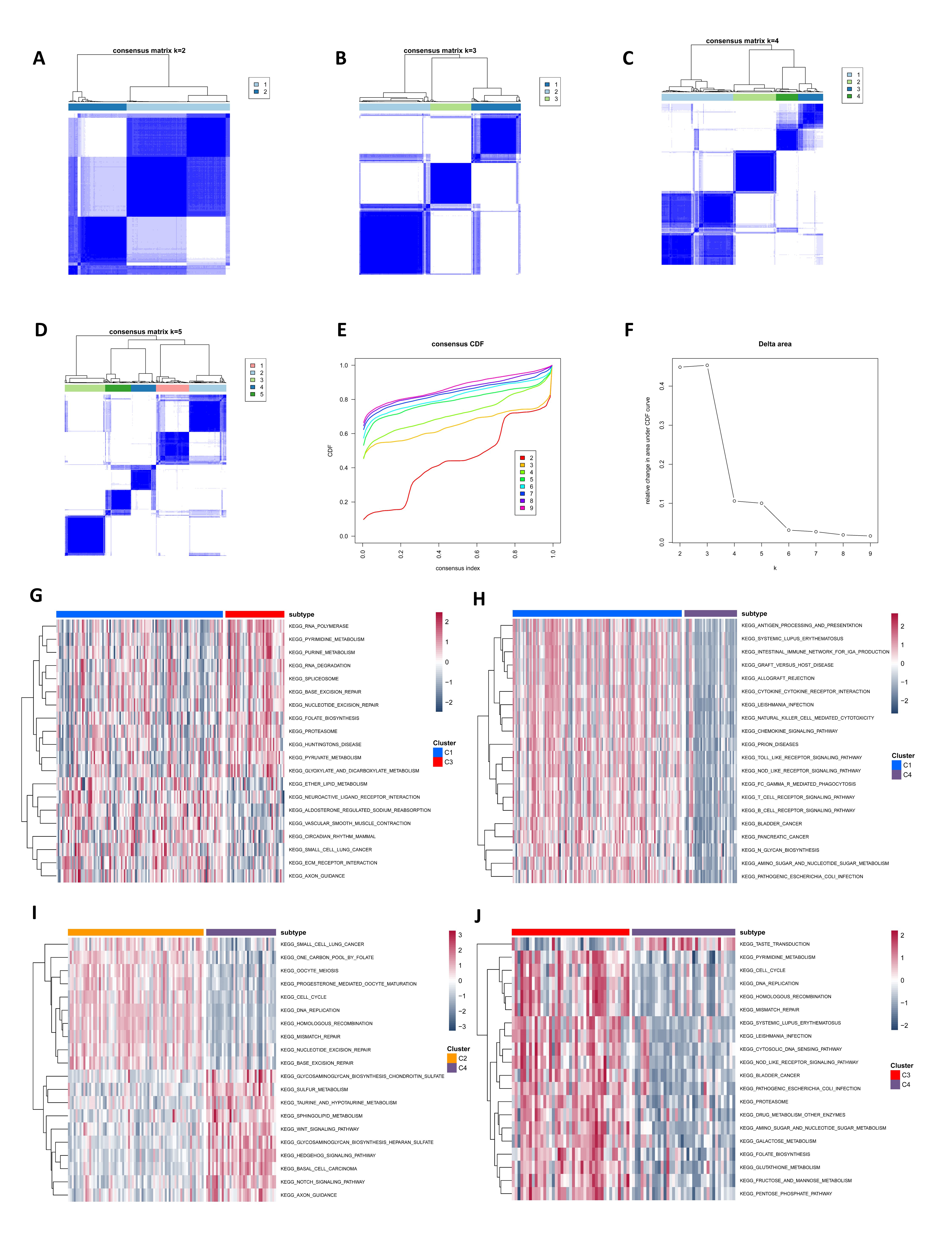

Supplement: Supplementary file 4 [file Image2.JPEG]
